# Intrinsic nonlinear dynamics drive single-species systems

**DOI:** 10.1073/pnas.2209601119

**Published:** 2022-10-24

**Authors:** Johannes Werner, Tobias Pietsch, Frank M. Hilker, Hartmut Arndt

**Affiliations:** ^a^Department of General Ecology, Institute of Zoology, University of Cologne, D-50674 Cologne, Germany;; ^b^Institute of Mathematics and Institute of Environmental Systems Research, School of Mathematics/Computer Science, Osnabrück University, D-49076 Osnabrück, Germany

**Keywords:** nonlinear dynamics, chaos, theory, chemostat, population ecology

## Abstract

The importance of oscillations and deterministic chaos in natural biological systems has been discussed for several decades and was originally based on discrete-time population growth models (May 1974). Recently, all types of nonlinear dynamics were shown for experimental communities where several species interact. Yet, there are no data exhibiting the whole range of nonlinear dynamics for single-species systems without trophic interactions. Up until now, ecological experiments and models ignored the intracellular dimension, which includes multiple nonlinear processes even within one cell type. Here, we show that dynamics of single-species systems of protists in continuous experimental chemostat systems and corresponding continuous-time models reveal typical characteristics of nonlinear dynamics and even deterministic chaos, a very rare discovery. An automatic cell registration enabled a continuous and undisturbed analysis of dynamic behavior with a high temporal resolution. Our simple and general model considering the cell cycle exhibits a remarkable spectrum of dynamic behavior. Chaos-like dynamics were shown in continuous single-species populations in experimental and modeling data on the level of a single type of cells without any external forcing. This study demonstrates how complex processes occurring in single cells influence dynamics on the population level. Nonlinearity should be considered as an important phenomenon in cell biology and single-species dynamics and also, for the maintenance of high biodiversity in nature, a prerequisite for nature conservation.

Simple models of population growth can show unpredictable and aperiodic behavior driven by intrinsic mechanisms (1). This led to an intensive debate on whether natural systems are characterized by chaotic behavior and how widespread chaotic dynamics are (2–6). In this context, the term “deterministic chaos” is defined as aperiodic fluctuations with sensitive dependence on the initial conditions (2, 5). Experimental evidence for the existence of chaos in populations is still rare due to several reasons. Empirical data can be composed of both deterministic and stochastic parts (5). Highly controlled laboratory experiments reducing contaminating external perturbations proved to be a sensitive method for detecting intrinsic mechanisms (3, 4). Up until now, chaos-like oscillations without external disturbance have not yet been identified in continuous single-species populations either in real world experiments or to our knowledge, in models.

A general assumption in ecology is that nonlinear dynamics originate from interaction processes, species, or cell types (2, 7). Major events of each eukaryotic cell cycle are regulated by a complex network of biochemical processes interacting within one cell, mostly controlled by different cytokines (Fig. 1*A*) (8, 9). Nonlinearity and its consequences have been discussed (8); however, they have seldom been considered regarding the dynamic behavior of cells (10). Biochemical processes are driven by changes in concentrations of biochemical products causing ups and downs in regulatory pathways (9); their oscillations are never exactly repeated (8). Intrinsic oscillations of fast-growing microbial species would allow a high diversity (6, 7) as an important prerequisite for maximum productivity and system stability (11), and this knowledge would have fundamental consequences for understanding key processes, allowing the coexistence of larger species and maintaining a high biodiversity in nature (6, 11). We hypothesized that the dynamics of one cell type in the absence of external disturbances should show unforeseeable dynamics, including chaos-like oscillations.

**Fig. 1.**
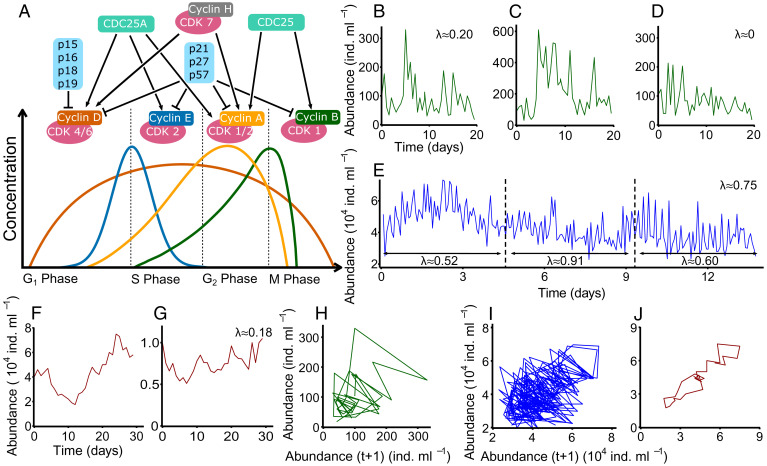
Nonlinear dynamics of single-species systems. (*A*) Schematic summary of the present understanding of changes in expression levels of eukaryote cyclins throughout the cell cycle. Cyclin-dependent kinases (CDKs) stimulate the development through the cell cycle and are positively regulated by cyclins and negatively regulated by CDK inhibitors (p15, p16, p18, p19, p21, p27, p57). All cyclin curves show nonlinear behavior. (*B*–*D*) Experimental results of abundance dynamics in bacteria-free chemostat systems (well-controlled flow-through systems) with the stramenopile flagellates *P. malhamensis* (dilution rates: 0.2 d^−1^ [*B*], 0.4 d^−1^ [*C*], and 0.2 d^−1^ [*D*]). (*E*) Experimental results of abundance dynamics in bacteria-free chemostats with the stramenopile flagellate *C. danica* (dilution rate: 0.2 d^−1^). (*F* and *G*) Literature data (15) on the undisturbed abundance dynamics of the planktonic diatom *Synedra* sp. (*F*) and *F. crotonensis* (*G*). Lyapunov exponents are given when they could be estimated. (*F*) Adapted from ref. 15. (*G*) Adapted from ref. 13. (*H*–*J*) Time delay reconstructions of datasets in *B*, *E*, and *F*, respectively (color coded correspondingly). Abundance is given in individuals (ind.) per ml.

## Results and Discussion

### Aperiodic Fluctuations in Single-Species Systems.

We established bacteria-free chemostat systems (well-controlled flow-through systems) with the stramenopile flagellates *Poterioochromonas malhamensis* (Fig. 1 *B*–*D* and Dataset S1) and *Chlorochromonas danica* (Fig. 1*E* and Dataset S2) at different dilution rates. These experiments with the unicellular eukaryotes allow for the analysis of intrinsic dynamics without any external disturbance (*SI Appendix*) and provide conclusive insights into dynamics that are difficult to derive from larger natural systems (4). While ecological theory states that intrinsic population mechanisms are much more likely to lead to stable dynamics or first-order cycles (2, 5), we identified aperiodic (chaos-like) fluctuations for nearly all experimental systems, a rare discovery in experimental systems. Due to the limited amount of data available for empirical studies, distinguishing deterministic dynamics [chaos, stable limit cycles, damped oscillations (2)] from noise, only approximations are possible (*SI Appendix*). Two experiments (Fig. 1 *B* and *E*) revealed positive Lyapunov exponents indicating chaos-like dynamics; one experiment (Fig. 1*D*) showed values near zero, indicating stable limit cycles, and for one experiment (Fig. 1*C*), the Lyapunov exponent could not be robustly determined. Two experiments had the same flow rate (Fig. 1 *B* and *D*); the different Lyapunov exponents could originate from temporary oscillations, multistability, or transient effects of initial conditions. The comparatively long time series (in comparison with other studies in the literature) available for the *Chlorochromonas* chemostat system allowed for a split of the data, which in addition to the total dataset, still revealed positive Lyapunov exponents (Fig. 1*E*). This demonstrates that even subsets of our time series show characteristics of chaotic dynamics. Laboratory experiments with three-species systems showed that the dynamic behavior may change at small changes in experimental conditions (4, 12, 13). We assume that such changes in experimental conditions might also cause not only chaotic and cyclic but also, damped oscillations. Only mathematical models could provide enough data to analyze the dynamic behavior more accurately.

### Chaos-Like Dynamics in a Continuous-Time Model.

**Fig. 2.**
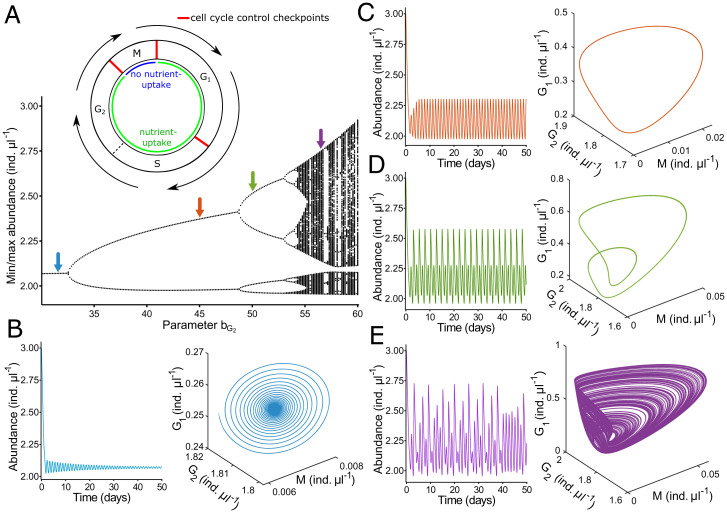
Simulations of the single-species chemostat model. (*A*) Bifurcation diagram using the dependence on the growth rate of cells in the G2 phase as an example. Arrows are related to the corresponding graphs in *B, C*, and *D*.*Inset* illustrates the model structure showing the cell cycle phases and model assumptions based on refs. 8 and 15. . Inset is modified from ref. 14 (© 1997 by the Ecological Society of America) and ref. 16. (*B*–*D*) Total abundance over time (*Left*) and in phase space (*Right*), resulting in (*B*) damped oscillations, (*C*) stable limit cycles (two points), (*D*) stable limit cycles (four points), and chaotic dynamics. Abundance is given in individuals (ind.) per µl.

Thus far, all single-species models exhibiting chaos relied on discrete-time structure or external forcing (1, 3, 14). However, since chemostat populations are generationally overlapping and continuously growing, continuous-time systems are better suited for modeling and explaining their population dynamics (5). To analyze the dynamics of our single-species systems, we applied the general cell cycle model known to be similar for all eukaryotes with its different phases (G1, S, G2, and M), which can be modulated by nutrients (Fig. 1*A*) (e.g., refs. 8, 9, and 14). Even though most of the cell cycle control is conserved in all eukaryotes (8, 9), in contrast to yeast, flagellated protists like many other cell types do not take up nutrients during the M phase (15). Using the basic principles of cell biology, we established a simple continuous-time mathematical model (*SI Appendix*) with the aim to uncover the whole range of qualitative system dynamics (proof of principle). Using a baseline set of parameter values, we calculated a bifurcation diagram depending on the growth rate of the G2 phase, indicating a period-doubling route to chaos (Fig. 2*A*). A similar principal behavior for the same parameter set would be obtained when using the dilution rate as a bifurcation parameter. This simple model showed all types of dynamic behavior by modifying a single parameter of the cell cycle (Fig. 2 *B*–*D*). Based on structured variables, the model can be analyzed for different functional forms of maturation rate, uptake rate, and cell division. It can simulate the abundance of nearly every unicellular eukaryote for periods of asexual reproduction.

### Aperiodic Fluctuations Could Be More Widespread than Previously Thought.

There are only a very few studies other than our study that documented long-term dynamics at undisturbed conditions in single-cell systems. The dynamics of the planktonic diatoms *Synedra* sp. and *Fragilaria crotonensis* (13) (Fig. 1 *F* and *G*) showed oscillating abundances with positive Lyapunov exponents and a bounded but not converging time delay reconstruction (Fig. 1*J*), indicating chaos-like dynamics in these single-species systems. Except for a few studies (e.g., refs. 10 and 13), datasets obtained from experimental time series are usually too short so that potentially chaotic dynamics cannot be found. This is of special importance since oscillations are crucial for the coexistence of species and allow for a high species diversity (e.g., ref. 6).

### Conclusion.

Our experimental and model results show characteristics of deterministic nonlinear dynamics, including chaos-like oscillations. The theoretical model establishes mechanisms of nonlinear interactions in single-species systems in general. In contrast to other single-species studies (e.g., refs. 3, 10, and 14), we used constant external conditions and no forced cell synchronization for the model and for the experiments. It is a continuous-time model of a single species that can show deterministic chaos without external forcing. The “zooming in” from single-species populations to intracellular processes provides explanations for the appearance of intrinsic nonlinear dynamics and will have an impact on the determination and understanding of population dynamics and cell–cell interactions. The phenomenon also has fundamental consequences for understanding evolutionary processes with the potential coexistence of competing species or cell lines at oscillating abundances (6), a basis for the high biodiversity on Earth. Maintaining these oscillations is essential for protecting biodiversity and its functions (11).

## Materials and Methods

### Continuous Cultivation in Chemostat Experiments.

To study intrinsically driven dynamics of protist populations, bacteria-free chemostat experiments (4, 12) were carried out at 20 °C in fully controlled and constant external conditions. Chemostats inoculated with the heterotrophic flagellate *P. malhamensis* were run at dilution rates of 0.2, 0.4, or 0.2 per day (Fig. 1 *B*–*D*). Samples were automatically taken every 12 h and microscopically analyzed (38 measurements). Another bacteria-free chemostat system was run in the dark with the mixotrophic flagellate *C. danica* at a dilution rate of 0.2 per day (Fig. 1*E*) and sampled using a newly developed automatic single-cell registration by noninvasive video microscopy (220 measurements). Details of all materials and methods are provided (*SI Appendix*).

### Model of the Cell Cycle.

The mathematical model extends established cell cycle models by implementing characteristics of dynamics of cell abundance in chemostat systems. The model uses the distinction of three stages of the cell cycle following the cell cycle control stages of eukaryotes (8). The first stage describes immature cells after cell division (G1 stage), the second considers mature cells in and after the synthesis stage (S and G2 stages), and the third stage describes cells during cell division (M stage). The model consists of four differential equations. Details of the model are provided (*SI Appendix*).

## Supplementary Material

Supplementary File

Supplementary File

Supplementary File

## Data Availability

All data generated or analyzed during this study are included in the article and/or supporting information.
