# Supplementary material for: Intrinsic nonlinear dynamics drive single-species systems
Source: Proc Natl Acad Sci U S A. 2022 Oct 24;119(44):e2209601119. doi: 10.1073/pnas.2209601119 (PMC9636902; doi:10.1073/pnas.2209601119)
Supplement: Supplementary File [file pnas.2209601119.sd01.pdf]

Dataset S1

Abundances in bacteria-free *Poterioochromonas malhamensis* chemostats

chemostat	flow rate	time (days)	abundance (ind/ml)
1	0.2	0	103.2301032
1	0.2	0.5	176.5234765
1	0.2	1	59.97002997
1	0.2	1.5	33.33333333
1	0.2	2	93.27672328
1	0.2	2.5	63.2967033
1	0.2	3	43.2967033
1	0.2	3.5	59.94005994
1	0.2	4	73.28005328
1	0.2	4.5	99.91341991
1	0.2	5	329.7935398
1	0.2	5.5	156.5334665
1	0.2	6	216.6433566
1	0.2	6.5	89.98667999
1	0.2	7	176.6000666
1	0.2	7.5	43.3033633
1	0.2	8	93.29004329
1	0.2	8.5	49.97335997
1	0.2	9	89.97335997
1	0.2	9.5	29.98334998
1	0.2	9	113.2933733
1	0.2	10.5	89.98001998
1	0.2	11	46.66666667
1	0.2	11.5	56.65667666
1	0.2	12	39.97668998
1	0.2	12.5	82.44255744
1	0.2	13	187.4050949
1	0.2	13.5	102.4250749
1	0.2	14	32.47252747
1	0.2	14.5	34.995005
1	0.2	15	179.8326673
1	0.2	16	67.45504496
1	0.2	16.5	107.5
1	0.2	17	77.46003996
1	0.2	17.5	44.95504496
1	0.2	18	99.94505495
1	0.2	18.5	77.44505495
1	0.2	19	42.48501499
1	0.2	19.5	17.48751249
1	0.2	20	29.97002997
2	0.4	0	89.98334998
2	0.4	0.5	116.5800866
2	0.4	1	126.5601066
2	0.4	1.5	29.98667999

2	0.4	2	173.1601732
2	0.4	2.5	63.31002331
2	0.4	3	36.64335664
2	0.4	3.5	56.61005661
2	0.4	4	163.2767233
2	0.4	4.5	609.5104895
2	0.4	5	413.030303
2	0.4	5.5	366.1509881
2	0.4	6	503.1901432
2	0.4	6.5	236.5334665
2	0.4	7	352.980353
2	0.4	7.5	526.1405261
2	0.4	8	519.6803197
2	0.4	8.5	256.4935065
2	0.4	9	276.6000666
2	0.4	9.5	229.9100899
2	0.4	9	296.3702964
2	0.4	10.5	189.8468198
2	0.4	11	476.9845219
2	0.4	11.5	173.2167832
2	0.4	12	133.1735463
2	0.4	12.5	67.45254745
2	0.4	13	164.955045
2	0.4	13.5	109.9225774
2	0.4	14	64.96503497
2	0.4	14.5	147.3526474
2	0.4	15	104.8951049
2	0.4	16	432.1903097
2	0.4	16.5	119.8801199
2	0.4	17	174.8251748
2	0.4	17.5	142.3502196
2	0.4	18	164.8801199
2	0.4	18.5	124.9175824
2	0.4	19	187.3701299
2	0.4	19.5	74.95504496
2	0.4	20	174.9725275
3	0.2	0	106.5601066
3	0.2	0.5	123.2434232
3	0.2	1	39.96003996
3	0.2	1.5	39.98001998
3	0.2	2	213.3233433
3	0.2	2.5	69.94338994
3	0.2	3	206.6666667
3	0.2	3.5	33.31002331
3	0.2	4	69.96503497
3	0.2	4.5	119.9267399
3	0.2	5	203.2467532
3	0.2	5.5	83.25008325
3	0.2	6	83.3033633

3	0.2	6.5	33.31002331
3	0.2	7	69.98001998
3	0.2	7.5	49.95004995
3	0.2	8	133.2001332
3	0.2	8.5	66.61671662
3	0.2	9	83.25008325
3	0.2	9.5	109.99001
3	0.2	9	96.63336663
3	0.2	10.5	66.63669664
3	0.2	11	129.9234099
3	0.2	11.5	33.32001332
3	0.2	12	99.93006993
3	0.2	12.5	54.95504496
3	0.2	13	97.47752248
3	0.2	13.5	79.995005
3	0.2	14	54.97002997
3	0.2	14.5	37.46753247
3	0.2	15	47.48501499
3	0.2	16	97.42007992
3	0.2	16.5	64.95257736
3	0.2	17	52.44755245
3	0.2	17.5	47.48251748
3	0.2	18	77.43756244
3	0.2	18.5	39.97502498
3	0.2	19	57.495005
3	0.2	19.5	17.49250749
3	0.2	20	63.2967033
