# Supplementary material for: Intrinsic nonlinear dynamics drive single-species systems
Source: Proc Natl Acad Sci U S A. 2022 Oct 24;119(44):e2209601119. doi: 10.1073/pnas.2209601119 (PMC9636902; doi:10.1073/pnas.2209601119)
Supplement: Supplementary File [file pnas.2209601119.sd02.pdf]

Dataset S2

Abundances in bacteria-free *Chlorochromonas danica* chemostat

Time (days)	Abundance (ind/ml)
0.02	6281
0.08	5898
0.15	5946
0.21	6334
0.27	6802
0.33	7059
0.4	7392
0.46	7672
0.52	8159
0.58	8759
0.65	8750
0.71	7465
0.77	5303
0.83	4770
0.9	4650
0.96	5610
1.02	7762
1.08	7825
1.15	8289
1.21	7655
1.27	8397
1.33	8326
1.4	8775
1.46	8055
1.52	9072
1.58	8497
1.65	5109
1.71	9941
1.77	5893
1.83	6360
1.9	4993
1.96	6195
2.02	8045
2.08	7829
2.15	8384
2.21	6481
2.27	6432
2.33	5352
2.4	8873
2.46	5915
2.52	6206

2.58	6792
2.65	7094
2.71	6502
2.77	9581
2.83	8925
2.9	6558
2.96	8027
3.02	8245
3.08	6440
3.15	6576
3.21	5181
3.27	5907
3.33	5749
3.4	4364
3.46	4211
3.52	5272
3.58	4690
3.65	5053
3.71	4142
3.77	6027
3.83	4857
3.9	5806
3.96	6575
4.02	5245
4.08	6444
4.15	6049
4.21	5292
4.27	6668
4.33	4942
4.4	4428
4.46	4538
4.52	4134
4.58	5466
4.65	4354
4.71	3894
4.77	3953
4.83	4618
4.9	4495
4.96	5224
5.02	4483
5.08	4532
5.15	3821
5.21	4178
5.27	4335
5.33	3839
5.4	3951
5.46	2895
5.52	3101
5.58	3106

5.65	3077
5.71	3165
5.77	3044
5.83	3520
5.9	3368
5.96	3531
6.02	4332
6.08	4456
6.15	4355
6.21	4286
6.27	3804
6.33	3837
6.4	3412
6.46	3041
6.52	3284
6.58	3469
6.65	3869
6.71	2945
6.77	2872
6.83	3162
6.9	2487
6.96	2255
7.02	3087
7.08	1501
7.15	2438
7.21	2740
7.27	3062
7.33	2424
7.4	2208
7.46	3392
7.52	2984
7.58	2825
7.65	2293
7.71	3943
7.77	3288
7.83	2719
7.9	3576
7.96	2947
8.02	3019
8.08	3536
8.15	4023
8.21	3161
8.27	2814
8.33	3271
8.4	3115
8.46	2915
8.52	4240
8.58	3193
8.65	4222

8.71	3167
8.77	3756
8.83	2564
8.9	3581
8.96	3010
9.02	3406
9.08	2866
9.15	3621
9.21	4392
9.27	4343
9.33	3189
9.4	3442
9.46	4344
9.52	4209
9.58	3194
9.65	3170
9.71	4162
9.77	3166
9.83	3491
9.9	2965
9.96	2466
10.02	3368
10.08	3680
10.15	2718
10.21	3199
10.27	3030
10.33	3335
10.4	2998
10.46	2511
10.52	2344
10.58	3074
10.65	2510
10.71	2928
10.77	2604
10.83	1875
10.9	3071
10.96	3793
11.02	2701
11.08	2634
11.15	2558
11.21	2868
11.27	2246
11.33	2595
11.4	2644
11.46	2639
11.52	2283
11.58	3122
11.65	2574
11.71	2591

11.77	3062
11.83	2152
11.9	2455
11.96	2714
12.02	3239
12.08	2427
12.15	2874
12.21	3193
12.27	2646
12.33	2512
12.4	3153
12.46	2319
12.52	2281
12.58	2792
12.65	2512
12.71	1944
12.77	2611
12.83	2814
12.9	1972
12.96	3032
13.02	2160
13.08	2301
13.15	2695
13.21	3751
13.27	3005
13.33	3634
13.4	2101
13.46	2175
13.52	2305
13.58	2329
13.65	2186
13.71	2040
13.77	2668
13.83	2007
13.9	2709
13.96	1748
14.02	2873
14.08	2053
14.15	2115
14.21	2199
14.27	2188
14.33	2170
14.4	2272
14.46	1917
14.52	2565
14.58	1372
14.65	2775
14.71	1698
14.77	1850

14.83	2253
14.9	2689
14.96	1969
15.02	2191
15.08	3172
15.15	2055
15.21	2123
15.27	1949
15.33	2222
15.4	1875
15.46	2113
15.52	1909
15.58	1730
15.65	2478
15.71	2031
15.77	1802
15.83	3392
15.9	1625
15.96	3048
16.02	2803
16.08	2053
16.15	3198
16.21	2879
16.27	2205
16.33	2614
16.4	3232
16.46	2212
16.52	2177
16.58	3753
16.65	2001
16.71	2127
16.77	3904
16.83	2260
16.9	2259
16.96	2182
17.02	3631
17.08	1844
17.15	2154
17.21	2146
17.27	3292
17.33	2207
17.4	1920
17.46	1400
17.52	3600
17.58	1560
17.65	1957
17.71	2128
17.77	2474
17.83	1636

17.9	1748
17.96	2658
18.02	1799
18.08	1901
18.15	2069
18.21	2167
18.27	2393
18.33	1397
18.4	2426
18.46	1708
18.52	2019
18.58	3571
18.65	1582
18.71	1752
18.77	2765
18.83	2078
18.9	1770
18.96	1763
19.02	2735
19.08	2284
19.15	1819
19.21	2643
19.27	1678
19.33	1681
19.4	1847
19.46	3101
19.52	1796
19.58	1517
19.65	1873
19.71	2898
19.77	1949
19.83	2152
19.9	2346
19.96	2228
20.02	3095
20.08	1974
20.15	2571
20.21	2485
20.27	2559
20.33	1826
20.4	1798
20.46	2183
20.52	2355
20.58	2007
20.65	1758
20.71	1495
