# Supplementary material for: Intrinsic nonlinear dynamics drive single-species systems
Source: Proc Natl Acad Sci U S A. 2022 Oct 24;119(44):e2209601119. doi: 10.1073/pnas.2209601119 (PMC9636902; doi:10.1073/pnas.2209601119)
Supplement: Supplementary File [file pnas.2209601119.sapp.pdf]

Supplementary Information for

Intrinsic non-linear dynamics drive single-species systems

Johannes Werner, Tobias Pietsch, Frank M. Hilker, Hartmut Arndt

corresponding author: Hartmut Arndt

Email: Hartmut.Arndt@uni-koeln.de

This PDF file includes:

Supplementary text

SI References

Supplementary text

The chemostat system. Chemostat experiments were carried out with two different stramenopile flagellates. We established axenic cultures of the flagellates *Poterioochromonas malhamensis* (HFCC 75, Heterotrophic Flagellate Culture Collection Cologne) and *Chlorochromonas danica* (provided by the Culture Collection of Algae at the University of Göttingen, Germany (SAG), type strain SAG 933-7) in 185 ml sterile one-stage glass reactors at 20°C in an air-conditioned room in the light after Becks et al. (1). All chemostats were surrounded by a water bath to minimize temperature fluctuations. All chemostat experiments were performed at the same experimental setup under the same constant external conditions. As inorganic nutrition for *Chlorochromonas danica* chemostats, WC minimal medium with 1.5 g/l glucose was used. Chemostat cultures of *Poterioochromonas malhamensis* were supplied with 90% WC and 10% SAG (glucose, proteose peptone, and yeast extract at a concentration of 1 g/l). Constant procedures of dilution (and sampling) were established by automated syringe pumps (Cavro XLP 6000, Modular Syringe Pump, TECAN, Crailsheim, Germany). *Poterioochromonas* was sampled by a computer-controlled sampling robot (RSP 9000 Cavro, TECAN, Crailsheim, Germany, triplicate samples, 1 ml each) with sampling intervals of 12 hours and was directly fixed in Lugol's solution (iodine-potassium iodide solution). The *Chlorochromonas* chemostat system was sampled using a newly developed continuous cell registration by non-invasive video-microscopy (220 measurements). *Chlorochromonas danica* chemostats were sampled every 1.5 hours via video analysis through a small channel (µ-slides V I0.1, IBIDI GmbH, Gräfelfing, Germany). Organisms were automatically detected through contour and movement detection. Each video contained 60 µl of the sample with an average of about 3600 organisms. All chemostats were kept sterile aided by a series of sterile air filters and UV-light exposure of the pumps. Every day, chemostats were checked for contamination.

For an analysis of the dynamic behavior over the undisturbed time, different methods were used. The dynamic behavior of the time series data of the chemostat experiments was characterized by the largest corresponding Lyapunov exponent (λ) to quantify the exponential separations of initially close trajectories and by phase-space diagrams to identify patterns of temporal changes. Calculations were carried out using the tseriesChaos package (tseriesChaos 0.1-13.1, https://cran.r-project.org/package=tseriesChaos) and the algorithm of Kantz (2). The data points of the transient phase at the beginning of the experiments (days 1-10) were omitted for the analyses and plots. The Lyapunov exponent was estimated from the slope of a straight line fitted to the linear part of the *ln*-transformed divergence of the data. Depending on the species and chemostat, an embedding dimension between 3 and 8 was used. For validation, the robustness against higher embedding dimensions was tested. Positive estimates of the Lyapunov exponent point to chaos-like irregular dynamics, whereas negative values indicate stable solutions.

Two experiments revealed positive Lyapunov exponents indicating chaos-like dynamics, while one experiment (Fig. 1D) was characterized by a Lyapunov exponent of near to zero indicating stable limit cycles. The long time series available for the *Chlorochromonas* chemostat system allowed a split of the data set into thirds which all revealed positive Lyapunov exponents (Fig. 1F). However, depending on the method and experimental setup, chaotic dynamics are hard to distinguish from noise, stochasticity, and sampling error that could induce misinterpretations (3, 4). However, our chemostats were run under highly controlled conditions. Therefore, we argue that the measured dynamics were deterministic with low levels of noise offering strong experimental evidence for chaotic dynamics in a real-world single-species system. Laboratory experiments with three-species systems showed that the dynamic behavior may change between chaos-like, periodic, and damped dynamics depending on small changes in experimental conditions (5). Thus, we assume that different experimental conditions in our study might also cause not only aperiodic but also cyclic and damped oscillations.

In addition to estimating the Lyapunov exponent, we also used the 0-1 test for chaos by Gottwald and Melbourne in the modified version (6), which has been shown to be able to cope with moderate measurement noise (6). The modified 0-1 test predicted chaotic dynamics in all chemostat experiments (Fig. 1 B-E). We decided to report the results from the direct Lyapunov exponent estimations, because they seem to be more conservative on the one hand and more informative than a binary distinction as in the 0-1 test on the other hand.

The model system. Our experimental results showed that the known models were not capable to explain all observable population dynamics. Various modifications of the classic Monod model were published in the last decades trying to explain experimental results by implementing a continuous position of cells along the cell cycle (7-9) or by using a discrete variable describing changes of the position within the cell cycle (in two states, nutrient-dependent and -independent phases; 10). But none of these models exhibited chaotic dynamics without external forcing. Major events of the eukaryotic cell cycle are regulated by a complex network of biochemical processes interacting within one cell, mostly controlled by different cytokines (Fig. 1A)(11) and separated into four different stages. The first stage describes immature cells after cell division (G_1-stage), the second stage describes cells which are in the phase of DNA duplication (S-stage), the third stage (G_2-stage) describes mature cells preparing for the last stage M, where mitosis is taking place. Due to the cell cycle control system, the S- and G_2-stage were merged (11). In contrast to previous models, our model uses a physiologically distinct phase of the cell cycle (M phase) where cells stop nutrient uptake (12) expected to be typical for most unicellular eukaryotic cells as controlling molecules, their interactions, dynamics and systems biology of cell cycle control are largely conserved in these organisms (13,14).

The model consists of four ordinary differential equations. Three equations simulate the abundance of G_1, G_2 and M (cells/l), and one equation describes the nutrient concentration N in the chemostat (mass/l). The equations read

$$
\begin{aligned}
\frac{dG_1}{dt} &= -\beta_{G_1}(N)G_1 + 2zM - dG_1 \\
\frac{dG_2}{dt} &= \beta_{G_1}(N)G_1 - \beta_{G_2}(N)G_2 - dG_2 \\
\frac{dM}{dt} &= \beta_{G_2}(N)G_2 - zM - dM \\
\frac{dN}{dt} &= -\mu_{G_1}(N)G_1 - \mu_{G_2}(N)G_2 - dN + dN_{\mathrm{in}}
\end{aligned}
$$

The model comprises biological and physical processes. The physical part consists of the dilution rate, d and the nutrient input, N_{in}. The biological part includes the maturation/transfer processes from G_1 to G_2 with rate $\beta_{G_1}(N)$ and from G_2 to M with rate $\beta_{G_2}(N)$. Cell growth and maturation take place when the nutrient concentration is sufficiently large, but they also become saturated with increasing nutrient concentration. Therefore, both maturation rates are assumed sigmoidal functions of the form

$$\beta_i(N) = \frac{a_i N^{b_i}}{k_i + N^{b_i}}, \qquad a_i, k_i > 0, b_i > 1, i \in \{G_1, G_2\}.$$

The maturation processes are a fundamental prerequisite for transferring the cells into the next stage of the cell cycle. To simplify the model, only growing processes (G_1 and G_2) are considered to have a nutrient uptake, each of which follows a Monod function of the form

$$\mu_i(N) = \frac{m_i N}{c_i + N}, \qquad m_i, c_i > 0, i \in \{G_1, G_2\}.$$

Following Lemesle and Gouzé (10), the nutrient uptake rate function is different from the maturation rate because the energy used for growth is assumed to be different from the energy provided by the consumption of substrate. This means, e.g., that the cells need to reach a threshold in nutrient concentration before they invest energy into growth. The cell division is the process of the division of one mature cell into two immature cells. It is assumed to take place with a constant rate z.

Because of the difficulties measuring the abundances at each cell stage, it was not possible to estimate the parameters by fitting the model to the experimental data. Therefore, we set up the model as a proof of principle with the aim to obtain qualitatively different dynamical behavior, e.g. stable steady states, regular oscillations, and chaos. Nevertheless, we set values for the maturation rates (length of the phases G1 and G2) and the length of the mitosis phase to be biologically plausible. The parameters resulted in a duration of the M-phase of 16 min (z=90) which was obtained by Boenigk (12), a minimum of 3 hours for the G1-phase, and a minimum of 8 hours for

the G2-phase. The total duration of about 11 hours under non-limiting nutrient conditions matched the values we obtained from our observations in the chemostat system. Overall, the abundances in the model were in the same range as that of experimental data. These are the parameter values that were used for the calculations: $d = 0.3, N_{\text{in}} = 20, a_{G_1} = 12, a_{G_2} = 17, b_{G_1} = 30, b_{G_2} = \text{varied}, z = 90, k_{G_1} = 2,\ k_{G_2} = 60,\ m_{G_1} = 2,\ m_{G_2} = 6, c_{G_1} = 1, c_{G_2} = 1.$ The calculations were performed with Matlab Version R2021b (MathWorks, Inc., Matlab, Version R2021b, Natick, MA (2021), see Dataset S3).

SI References

1. L. Becks, L., F. M. Hilker, H. Malchow, K. Jürgens, H. Arndt, Experimental demonstration of chaos in a microbial food web. Nature 435, 1226-1229 (2005).
2. R. Hegger, H. Kantz, T. Schreiber, Practical implementation of nonlinear time series methods: The TISEAN package. Chaos: An Interdisciplinary Journal of Nonlinear Science 9, 413-435 (1999).
3. M. T. Rosenstein, J. J. Collins, C. J. De Luca, A practical method for calculating largest Lyapunov exponents from small data sets. Physica D: Nonlinear Phenomena 65, 117-134 (1993).
4. A. Hastings, C. L. Hom, S. Ellner, P. Turchin, H. C. J. Godfray, Chaos in ecology: is mother nature a strange attractor? Annual Review of Ecology and Systematics 24, 1-33 (1993).
5. L. Becks, H. Arndt, Transitions from stable equilibria to chaos, and back, in an experimental food web. Ecology 89, 3222-3226 (2008).
6. Gottwald, G. A. & Melbourne, I. Testing for chaos in deterministic systems with noise. Phys. D: Nonlinear Phenom. 212, 100–110 (2005).
7. M. Pascual, H. Caswell, H, From the cell cycle to population cycles in phytoplankton–nutrient interactions. Ecology 78, 897-912 (1997).
8. T. M. Massie, B. Blasius, G. Weithoff, U. Gaedke, G. F. Fussmann, Cycles, phase synchronization, and entrainment in single-species phytoplankton populations. Proceedings of the National Academy of Sciences 107, 4236-4241 (2010).
9. T. M. Massie, A. Ryabov, B. Blasius, G. Weithoff, U. Gaedke, Complex transient dynamics of stage-structured populations in response to environmental changes. The American Naturalist, 182, 103-119. (2013).
10. V. Lemesle, J. L. Gouzé, A simple unforced oscillatory growth model in the chemostat. Bulletin of Mathematical Biology 70, 344-357 (2008).
11. J. J. Tyson, A. Csikasz-Nagy, B. Novak, The dynamics of cell cycle regulation. Bioessays 24(12), 1095-1109 (2002).
12. J. Boenigk, Variability of ingestion rates with stage in cell cycle of a heterotrophic nanoflagellate (*Spumella* sp.) measured by an individual-based approach. European Journal of Protistology 38, 299-306 (2002).
13. D. O. Morgan, The Cell Cycle - Principles of Control, Oxford Univ Press, 297pp. (2007).
14. F.R. Cross, and J.G. Umen, The *Chlamydomonas* cell cycle. The Plant Journal *82*, 370-392 (2015).
